# The Food Contaminant Deoxynivalenol Exacerbates the Genotoxicity of Gut Microbiota

**DOI:** 10.1128/mBio.00007-17

**Published:** 2017-03-14

**Authors:** Delphine Payros, Ulrich Dobrindt, Patricia Martin, Thomas Secher, Ana Paula F. L. Bracarense, Michèle Boury, Joelle Laffitte, Philippe Pinton, Eric Oswald, Isabelle P. Oswald

**Affiliations:** a Toxalim (Research Centre in Food Toxicology), Université de Toulouse, INRA, ENVT, INP-Purpan, UPS, Toulouse, France; b Institute of Hygiene, University of Münster, Münster, Germany; c Interdisciplinary Center for Clinical Research (IZKF), University of Münster, Münster, Germany; d CHU Toulouse, Service de Bactériologie-Hygiène, Institut Fédératif de Biologie, TSA, Toulouse, France; e Institut de Recherche en Santé Digestive (IRSD), Université de Toulouse, INSERM, INRA, ENVT, UPS, CS, Toulouse, France; f Laboratorio Patologia Animal, CP, Universidade Estadual de Londrina, Londrina, Paraná, Brazil; University of British Columbia

## Abstract

An increasing number of human beings from developed countries are colonized by *Escherichia coli* strains producing colibactin, a genotoxin suspected to be associated with the development of colorectal cancers. Deoxynivalenol (DON) is the most prevalent mycotoxin that contaminates staple food—especially cereal products—in Europe and North America. This study investigates the effect of the food contaminant DON on the genotoxicity of the *E. coli* strains producing colibactin. *In vitro*, intestinal epithelial cells were coexposed to DON and *E. coli* producing colibactin. *In vivo*, newborn rats colonized at birth with *E. coli* producing colibactin were fed a DON-contaminated diet. Intestinal DNA damage was estimated by the phosphorylation of histone H2AX. DON exacerbates the genotoxicity of the *E. coli* producing colibactin in a time- and dose-dependent manner *in vitro*. Although DON had no effect on the composition of the gut microbiota, and especially on the number of *E. coli*, a significant increase in DNA damage was observed in intestinal epithelial cells of animals colonized by *E. coli* strains producing colibactin and coexposed to DON compared to animals colonized with *E. coli* strains unable to produce colibactin or animals exposed only to DON. In conclusion, our data demonstrate that the genotoxicity of *E. coli* strains producing colibactin, increasingly present in the microbiota of asymptomatic human beings, is modulated by the presence of DON in the diet. This raises questions about the synergism between food contaminants and gut microbiota with regard to intestinal carcinogenesis.

## IMPORTANCE

An increasing number of human beings from developed countries are colonized by *Escherichia coli* strains producing colibactin, a genotoxin suspected to be associated with the development of colorectal cancers. Deoxynivalenol (DON) is the most prevalent mycotoxin that contaminates staple food—especially cereal products—in Europe and North America. Our *in vitro* and *in vivo* results demonstrate that the intestinal DNA damage induced by colibactin-producing *E. coli* strains was exacerbated by the presence of DON in the diet. This raises questions about the synergism between food contaminants and gut microbiota with regard to intestinal carcinogenesis.

## INTRODUCTION

The gut is colonized by a rich ecological consortium of more than a thousand species of microorganisms that exert marked effects on basic host physiology, immunity, and metabolism (1–3). *Escherichia coli* bacteria are some of the pioneer bacteria that colonize the guts of mammals within a few days following birth (4). The genetic structure of the *E. coli* population is clonal and can be segregated into seven major phylogenetic groups (A, B1, B2, C, D, E, and F) (5). The prevalence of the B2 group is increasing among *E. coli* strains persisting in the microbiota of humans in developed countries (6, 7). This change in the distribution of phylogenetic groups of the *E. coli* population could be a consequence of enriched dietary habits and increased levels of hygiene in industrialized countries.

*E. coli* genomes show evidence of a widespread acquisition of functions through horizontal transfer of genes (8). Up to 50% of *E. coli* strains from the phylogenetic group B2 have acquired the *pks* genomic island (9, 10). This gene cluster encodes a nonribosomal peptide synthase-polyketide synthase (NRPS-PKS) assembly line and produces a genotoxic secondary metabolite called colibactin (9). A short contact between mammalian cells and *E. coli* producing colibactin induces DNA damage, senescence, and chromosomal abnormalities (9, 11–13). Colonization of the gut by phylogroup B2 *E. coli* producing colibactin is associated with the presence of DNA double-strand breaks in intestinal epithelial cells (14). Phylogroup B2 *E. coli* bacteria producing colibactin have an impact on host physiology (14, 15) and contribute to the development of colorectal cancer in mouse models of colitis (16, 17). Infants are colonized at birth with B2 *E. coli* expressing colibactin, and these *E. coli* strains have a long-term capacity to persist in the bowel microbiota (14, 18). Except for iron availability, little is known about the environmental factors that modulate the genotoxicity of these bacteria in the gut (19).

Mycotoxins are the most frequently occurring natural food contaminants in human and animal diet (20). Of the mycotoxins, deoxynivalenol (DON) is mainly produced by *Fusarium graminearum* and *Fusarium culmorum*. This toxin frequently develops in cereals and grains. A survey of 12 European countries indicated that 57% of samples were positive for DON (21). Analyses of urine samples lead to the estimation that 98% of adults in the United Kingdom had been exposed to DON, while 80% of children in the Netherlands exceeded the tolerable daily intake for this mycotoxin (22, 23). DON targets the intestine (24–26) and interacts with the peptidyl transferase region of the 60S ribosomal subunit, inducing a “ribotoxic stress,” resulting in the activation of mitogen-activated protein kinases (MAPKs) and their downstream pathways (27, 28).

The consequence of exposure to this food contaminant on the genotoxic potential of the gut microbiota has never been addressed. In the present study, we investigated the impact of DON on the genotoxicity of *E. coli* producing colibactin. Using both *in vitro* and *in vivo* experiments, we demonstrated that DON exacerbates the intestinal DNA damage induced by genotoxic strains of *E. coli*.

## RESULTS

### Genotoxicity of DON and colibactin-producing *E. coli* on intestinal epithelial cells.

**FIG 1:**
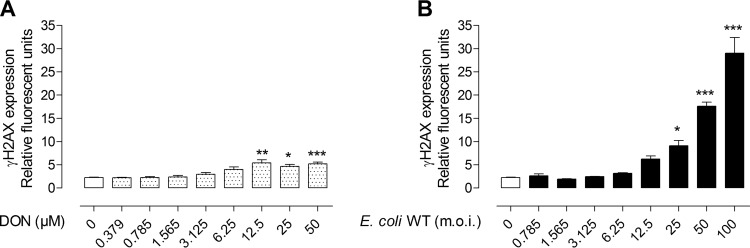
Quantification of DNA double-strand breaks in intestinal epithelial cells *in vitro*. (A) IEC-6 cells were treated with increasing doses of DON (0 to 50 µM) for 8 h, and γH2AX was quantified by an in-cell Western (ICW) method. Untreated cells (white bar) and cells exposed to DON (dotted white bars) are indicated. (B) IEC-6 cells were infected with wild-type *E. coli* producing colibactin (*E. coli* WT) for 4 h with increasing multiplicities of infection (MOIs [m.o.i. in the figure] of 0 to 100 bacteria per cell) before ICW analysis 4 h after infection (black bars). Mean values plus standard errors of the means (SEM) (error bars) from two independent experiments are shown. Values that are significantly different by one-way ANOVA with Bonferroni’s multiple-comparison correction are indicated by asterisks as follows: *, *P* < 0.05; **, *P* < 0.01; ***, *P* < 0.001.

The genotoxicity of DON and colibactin-producing *E. coli* was first examined in cultured rat intestinal epithelial cells (IEC-6) using an in-cell Western (ICW) technique that assessed the phosphorylated form of histone H2AX (γH2AX) as a marker of DNA double-strand breaks. DON alone did not exert detectable genotoxicity on IEC-6 cells, except at high doses (12.5 to 50 µM for at least 8 h) where low levels of γH2AX were observed (2.27 ± 0.22 relative fluorescence units [RFU] in control cells versus 4.64 ± 1.37 and 5.21 ± 1.30 RFU in cells treated with 25 µM and 50 µM DON, respectively [*P* < 0.05 and *P* < 0.001, respectively]) (Fig. 1A; see Fig. S1A and S2A in the supplemental material). In contrast to DON-treated cells, IEC-6 cells were highly susceptible to the genotoxicity of colibactin-producing *E. coli* (colibactin-producing wild-type *E. coli* [*E*. *coli* WT]). As shown in Fig. 1B, the infection of IEC-6 cells with *E. coli* WT induced a dose-dependent increase in γH2AX (2.27 ± 0.22 RFU in control cells versus 9.1 ± 3.92 RFU [*P* < 0.05] in *E. coli* WT with a multiplicity of infection [MOI] of 25 and 29.03 ± 8.19 RFU [*P* < 0.001] in *E. coli* WT with an MOI of 100 compared to noninfected cells).

10.1128/mBio.00007-17.2 FIG S1  (A) Experimental design of bacterial infection and exposure to DON *in vitro* on IEC-6 cells. The cells were incubated with increasing doses of DON (0 to 50 µM) and/or infected with *E. coli* strains (m.o.i. of 0 to 100) for 4 h. The cells were washed and incubated in cell culture medium supplemented with 200 µg ⋅ ml^−1^ gentamicin and maintained for 4 h postinfection in the presence or absence of DON before ICW, Western blot, or immunofluorescence analysis. (B) Design of rat experimental model. Evaluation of detailed *Enterobacteriaceae* gut colonization and *E. coli* strain colonization was done in tissue at PND 8 and in feces at PND 28, the time of weaning, and before exposure to DON and at PND 58 at the end of experiment after exposure to a DON-contaminated diet or no exposure to a DON-contaminated diet. DNA damage was analyzed in jejunal epithelial cells at the end of experiment (PND 58) by immunofluorescence analysis. The time points when the bacterial load was evaluated are indicated by an asterisk. The time point when histological score was evaluated and 16S microbiota analysis and immunofluorescence analysis were performed are indicated by a pound symbol. Download FIG S1, TIF file, 0.5 MB. Copyright © 2017 Payros et al. 2017 Payros et al. This content is distributed under the terms of the Creative Commons Attribution 4.0 International license.

10.1128/mBio.00007-17.3 FIG S2  Quantification of DNA double-strand breaks in cells exposed to DON or coinfected with *E. coli* producing colibactin after 4 h. Quantification of γH2AX expression was done by the ICW method. (A) IEC-6 cells were treated with increasing doses of DON (0 to 50 µM) for 4 h, and then γH2AX was quantified 4 h later. White bars represent untreated cells. Dotted white bars represent cells exposed to increasing doses of DON. (B) IEC-6 cells infected with *E. coli* WT or not infected with *E*. *coli* and treated with increasing doses of DON (0 to 50 µM) for 4 h. γH2AX was quantified 4 h later. Mean values ± SEM of two independent experiments are shown. ***, *P* < 0.001 by one-way ANOVA with Bonferroni’s multiple-comparison correction. Download FIG S2, TIF file, 0.1 MB. Copyright © 2017 Payros et al. 2017 Payros et al. This content is distributed under the terms of the Creative Commons Attribution 4.0 International license.

### DON exacerbates the genotoxicity of colibactin-producing *E. coli*.

**FIG 2:**
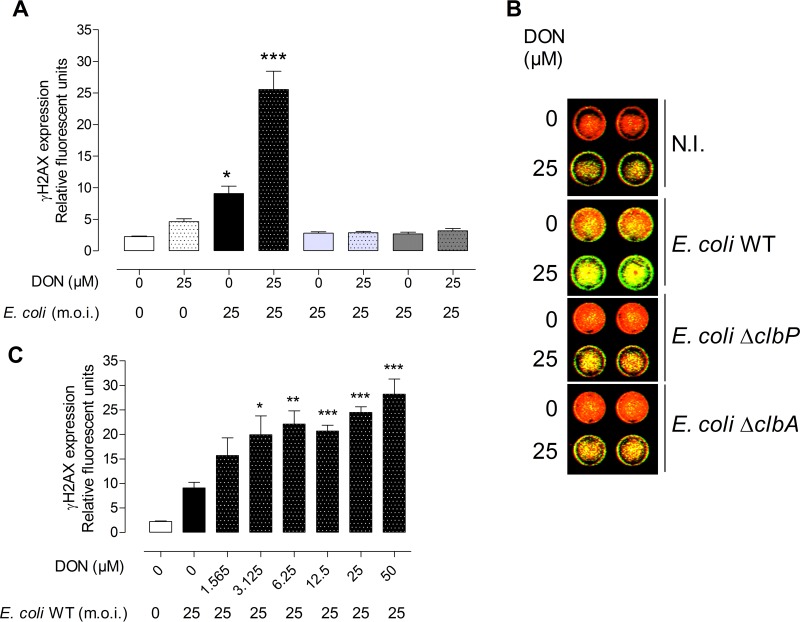
Dose-dependent synergistic genotoxicity of DON and *E. coli*. (A) IEC-6 cells infected for 4 h with *E. coli* strains producing colibactin (*E. coli* WT) or not producing colibactin (*E. coli* Δ*clbA* and *E. coli* Δ*clbP*) (MOI of 25) and coexposed to 25 µM DON 8 h before quantification of γH2AX by in-cell Western (ICW). (B) ICW pictures of IEC-6 cells exposed or not exposed to 25 µM DON and infected (MOI of 25) with *E. coli* strains producing colibactin (*E. coli* WT) or not producing colibactin (*E. coli* Δ*clbA* or Δ*clbP*). DNA is artificially colored red, and γH2AX is shown in green. N.I., not infected. (C) IEC-6 cells infected for 4 h with *E. coli* WT or not infected with *E*. *coli* and treated with increasing doses of DON (0 to 50 µM) 8 h before ICW. Control (not treated) cells (white bars) and cells infected with *E. coli* WT (black bars), *E. coli* Δ*clbP* (light gray bars), and *E. coli* Δ*clbA* (dark gray bars) are shown. Dotted white, black, light gray, and dark gray bars represent cells exposed to DON and coinfected with different *E. coli* strains (dotted black, WT; dotted light gray, Δ*clbP*; dotted dark gray, Δ*clbA*) or not coinfected with *E. coli* strains (dotted white). Mean values plus SEM from three independent experiments are shown. Values that are significantly different from the values for cells infected with colibactin-producing *E. coli* and exposed to DON with all other groups by one-way ANOVA with Bonferroni’s multiple-comparison correction are indicated by asterisks as follows: *, *P* < 0.05; **, *P* < 0.01; ***, *P* < 0.001.

To determine whether the genotoxic effect of colibactin can be modulated by exposure to the food contaminant DON, IEC-6 cells were infected with a low dose of *E. coli* WT (MOI of 25) and coexposed to DON (25 µM). Exposure of cells to DON exacerbated the genotoxicity of *E. coli* producing colibactin on IEC-6 cells compared to cells exposed solely to DON or infected solely by *E. coli* WT as demonstrated by the expression of γH2AX by an in-cell Western method (Fig. 2A and B) and Western blotting (data not shown). This exacerbation was dependent on the dose of DON (Fig. 2C) and was observed only when DON was present for 8 h during and after infection (Fig. S2B). The DNA double-strand breaks in intestinal cells was confirmed by the expression and quantification of p53 binding protein 1 (53BP1 [Fig. S3]) and was associated with an increased phosphorylation of the MAP kinase extracellular signal-regulated kinase 1 or 2 (ERK1/2) (data not shown).

10.1128/mBio.00007-17.4 FIG S3  Exposure to DON exacerbates the DNA double-strand breaks in rat IEC-6 cells *in vitro*. IEC-6 cells infected for 4 h with *E. coli* strains producing colibactin (*E. coli* WT) or not producing colibactin (*E. coli* Δ*clbA*) and coexposed to DON (25 µM) for 8 h or not coexposed to DON before immunofluorescence analysis. (A) DNA double-strand breaks were detected by γH2AX (green) and 53BP1 (red) immunostaining. Nuclei were stained with DAPI (blue). Bar = 20 µm. (B and C) Quantification of IEC-6 cells positive for γH2AX (B) and 53BP1 (C) focus formation. Cells were scored positive for focus formation when more than five foci or nuclei were observed. Mean values ± SEM of at least two independent experiments are shown. One-way ANOVA with Bonferroni’s multiple-comparison correction was used for statistical comparisons. Symbols: ***, *P* < 0.001 for cells infected with colibactin-producing *E. coli* and exposed to DON (8 h) compared to all other groups; ##, *P* < 0.01; ###, *P* < 0.001 for cells infected with colibactin-producing *E. coli* and exposed to DON (8 h) with cells infected with colibactin-producing *E. coli* and exposed to DON (0 or 4 h). Download FIG S3, TIF file, 1.4 MB. Copyright © 2017 Payros et al. 2017 Payros et al. This content is distributed under the terms of the Creative Commons Attribution 4.0 International license.

Using two independent *E. coli* mutants (*E. coli* Δ*clbA* and *E. coli* Δ*clbP*) that did not produce colibactin, we verified that the genotoxicity observed in the infected cells depended specifically on the production of colibactin. As shown in Fig. 2A, no genotoxicity was observed in IEC-6 cells infected with the mutants and exposed to DON. To verify that DON did not modulate the physiology or gene expression in *E. coli*, the effects of DON on bacterial growth and expression of the *pks* island genes were evaluated. Treatment of *E. coli* with increasing doses of DON for 24 h had no effect on bacterial growth, nor did it modify the expression of genes of the *pks* island coding for enzymes required for the production of colibactin (data not shown).

### Contamination of the diet with DON does not impair the colonization of the gut by *E. coli* strains producing colibactin or not producing colibactin.

**FIG 3:**
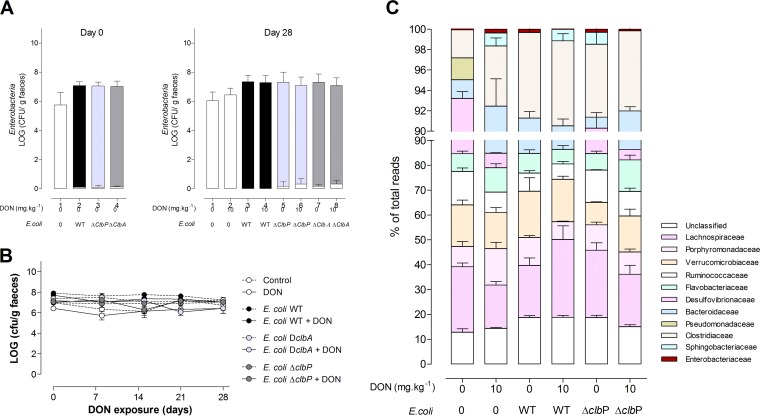
Exposure to DON does not impact intestinal colonization by *E. coli* and the overall composition of the intestinal microbiota. (A) Members of the family *Enterobacteriaceae* (white bar) and *E. coli* (*E. coli* WT [black bar], *E. coli* Δ*clbA* [light gray bar], and *E. coli* Δ*clbP* [dark gray bar]) were quantified in fecal homogenates at day 0 (postnatal day [PND] 28, the time of weaning) and day 28 (PND 58). (B) Quantification of *E. coli* in fecal homogenates after weaning (days 0 to 28) and exposure to a DON-contaminated diet (10 mg ⋅ kg^−1^) or no exposure to a DON-contaminated diet. Mean values ± SEM are shown (*n* = 9 or 10). D*clbA*, Δ*clbA*. (C) Evaluation of 16S microbiota diversity in adult animals (PND 58) colonized since birth by *E. coli* WT or *E. coli* Δ*clbP* or in animals in a control group and coexposed or not exposed to a DON-contaminated diet (10 mg ⋅ kg^−1^) (*n* = 4).

The next aim was to assess whether DON could exacerbate the genotoxicity exerted by colibactin-producing *E. coli* present in the gut microbiota. To this end, animals were colonized at birth with *E. coli* WT or *E. coli* Δ*clbA* and *E. coli* Δ*clbP* mutants. After weaning, the rats were fed for 4 weeks with a diet contaminated with DON or not contaminated with DON (Fig. S1B). We first examined the effect of DON on the colonization of the gut by *E. coli* and more generally on the microbiota. Ingestion of a DON-contaminated diet from weaning to adulthood did not modify the levels of enterobacteria in the feces compared to those in animals fed a normal diet or the fecal *E. coli* counts (Fig. 3A and B). A 16S microbiota analysis indicated that ingestion of the DON-contaminated diet did not significantly alter the composition or diversity of the gut microbiota of rats colonized at birth with *E. coli* strains producing colibactin or not producing colibactin (Fig. 3C).

### Exposure to DON reduces body weight gain and induces intestinal modifications in adult rats, independently of neonatal colonization by *E. coli* strains producing colibactin or not producing colibactin.

**FIG 4:**
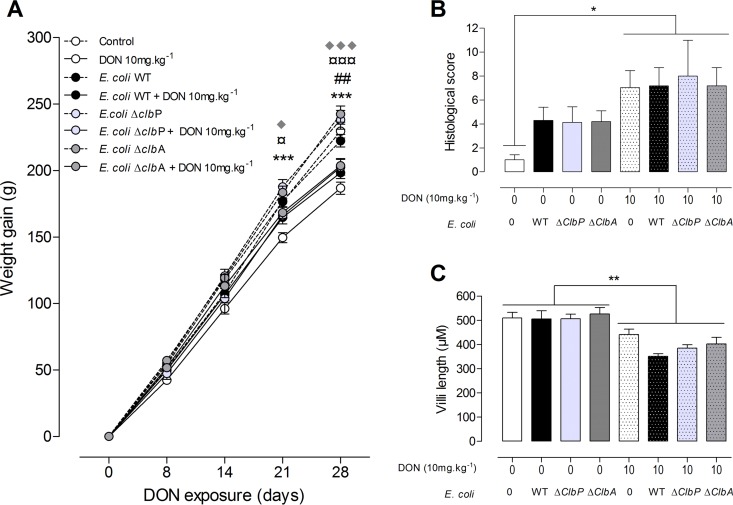
Exposure to DON reduces body weight gain and alters the jejunal tissue in adult animals independently of the colonizing *E. coli* strain. (A) Body weight gain was evaluated in the progeny from days 0 to 28. (B and C) Histological score (B) and villus length (C) in the jejuna of rats colonized since birth by *E. coli* strains producing colibactin or not producing colibactin or not colonized by *E*. *coli* and coexposed to a DON-contaminated diet (10 mg ⋅ kg^−1^) or not coexposed to a DON-contaminated diet. Mean values plus SEM are shown (*n* = 8 to 10). Values for animals fed a DON-contaminated diet (10 mg ⋅ kg^−1^) that are significantly different for the values for animals fed a control diet by one-way ANOVA with Bonferroni’s multiple-comparison correction are indicated by symbols as follows: * and ¤, *P* < 0.05; ## and **, *P* < 0.01; ***, ¤¤¤, and ♦♦♦, *P* < 0.0001. Values for control animals (*), animals colonized with *E. coli* WT (#), and animals colonized with the *E. coli* Δ*clb*P mutant (¤) or with the *E. coli* Δ*clb*A mutant (◆) are indicated.

As expected, ingestion of the DON-contaminated diet (10 mg ⋅ kg of body weight^−1^) significantly decreased body weight compared to animals fed a control diet (Fig. 4A). In addition, animal weight decreased independently of neonatal colonization with the *E. coli* strain producing colibactin or not producing colibactin. Similarly, as previously observed, in the jejuna of animals fed the DON-contaminated diet, increased histological alterations were observed, demonstrating moderate intestinal lesions and breakdown (Fig. 4B). A decreased villus height in the jejunum was also observed in these animals (Fig. 4C). These histological and morphological modifications occurred independently of colonization of animals by *E. coli* strains producing colibactin or not producing colibactin (Fig. 4B and C). These results suggest that colibactin-producing strains did not impact the classical effects of DON in relation to weight gain and morphology of the gut.

### Exposure to DON exacerbates the intestinal DNA damage induced by colibactin-producing *E. coli* in a dose- and time-dependent manner.

**FIG 5:**
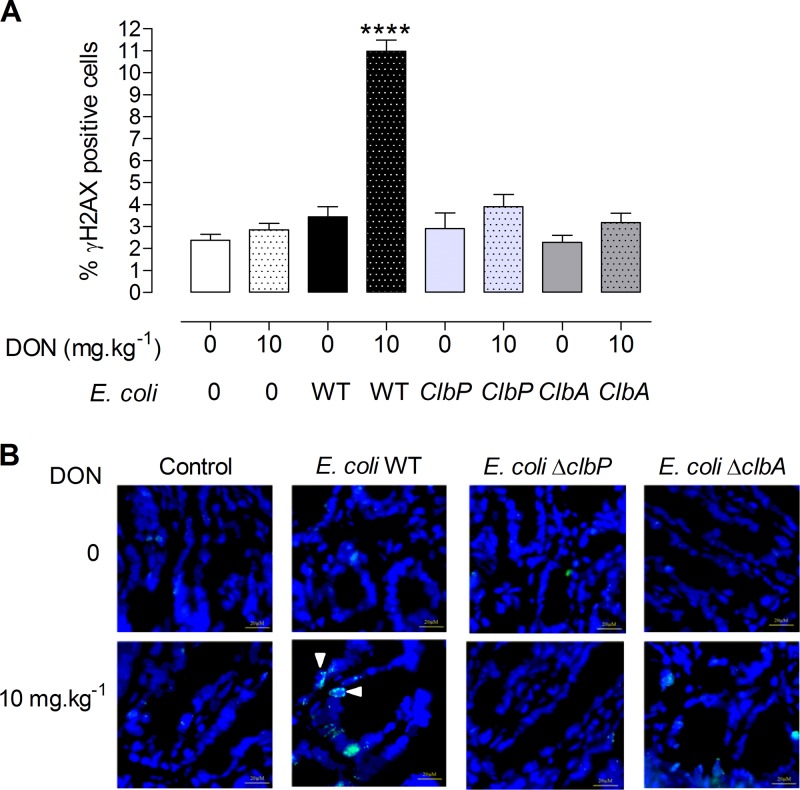
DON exacerbates DNA damage in jejunal epithelial cells of animals colonized by colibactin-producing *E. coli*. Immunofluorescence analysis of the jejunal epithelium of adults (PND 58) colonized since birth by *E*. *coli* strains (*E. coli* WT, *E. coli* Δ*clbA*, or *E. coli* Δ*clbP* strain) or treated with PBS (control group) and coexposed to a DON-contaminated diet (10 mg ⋅ kg^−1^) for 4 weeks or not coexposed to a DON-contaminated diet for 4 weeks. (A) Quantification of the percentage of γH2AX-positive cells in jejunal crypts. Mean values plus± SEM are shown (*n* = 8 to 10). Values that are significantly different (*P* < 0.0001) by one-way ANOVA with Bonferroni’s multiple-comparison correction are indicated by four asterisks. (B) Representative jejunal frozen sections at PND 58. DNA was stained in blue. γH2AX foci are shown in green. Bars = 10 µM.

The *in vivo* effect of a diet contaminated with DON and colibactin-producing *E. coli* on intestinal DNA damage was evaluated next. To this end, jejunal sections from animals colonized at birth with *E. coli* producing colibactin or not producing colibactin that were fed a control diet or a DON-contaminated diet for 4 weeks were stained for the phosphorylated form of H2AX. Dietary exposure of animals to 10 mg ⋅ kg^−1^ DON alone did not induce detectable DNA damage in intestinal epithelial cells (Fig. 5A, dotted white bar, and B). Similarly, adult rats colonized with *E. coli* strains producing colibactin and fed the control diet did not exhibit significant intestinal DNA damage (Fig. 5A, black bar). However, in animals colonized with *E. coli* WT and fed a diet contaminated with DON (10 mg ⋅ kg^−1^), a significant increase in γH2AX-positive epithelial cells was observed compared to rats exposed to DON or rats colonized with *E. coli* WT (Fig. 5A, dotted black bar, and B). As observed *in vitro*, no genotoxicity was observed in animals fed a DON-contaminated diet and colonized with non-colibactin-producing mutants (*E. coli* Δ*clbA* and *E. coli* Δ*clbP*) (Fig. 5A and B). The increase in DNA double-strand breaks observed in intestinal epithelial cells (IECs) of animals colonized since birth by colibactin-producing *E. coli* and exposed to a DON-contaminated diet was associated with a significant increase in activation of phosphorylated MAP kinase ERK p42/p44 (data not shown).

**FIG 6:**
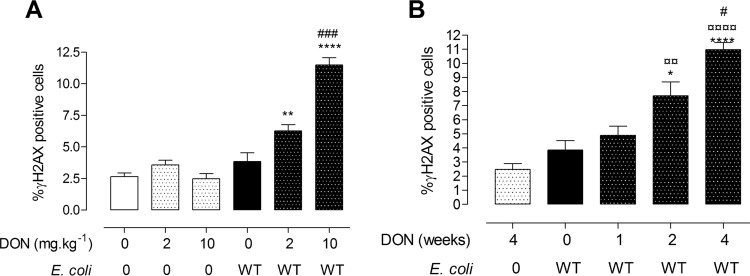
DON exacerbates DNA damage in jejunal epithelium in a dose- and time-dependent manner. Immunofluorescence analysis of jejunal epithelium was performed. (A and B) Quantification of γH2AX-positive cells in jejuna of animals exposed to different doses of DON (2 mg ⋅ kg^−1^ or 10 mg ⋅ kg^−1^) for 4 weeks and colonized or not since birth with *E. coli* WT (A) or exposed to DON (10 mg ⋅ kg^−1^) for 1, 2, or 4 weeks after weaning (B). Mean values plus SEM are shown (*n* = 6 to 10). Values that are significantly different for control animals (*) or DON-exposed animals (¤) versus *E. coli* WT-colonized animals exposed to DON by one-way ANOVA with Bonferroni’s multiple comparisons are indicated by symbols as follows: *, *P* < 0.05; ** and ¤¤, *P* < 0.01; **** and ¤¤¤¤, *P* < 0.000; ###, *P* < 0.001, group exposed to 2 mg ⋅ kg^−1^ of DON versus group exposed to 10 mg ⋅ kg^−1 ^of DON in panel A; #, *P* < 0.05, *E. coli* WT-colonized animals exposed to DON for 2 weeks versus *E. coli* WT-colonized animals exposed to DON for 4 weeks in panel B.

We next investigated the effect of the dose or duration of DON exposure on the genotoxic effect of colibactin. We first assessed the effect of the dose of DON on DNA damage in control animals or animals colonized with *E. coli* WT and fed diets contaminated with 2 or 10 mg ⋅ kg^−1^ DON (Fig. 6A). In rats exposed for 4 weeks to the lowest dose of DON contamination, exacerbation of DNA damage induced by the genotoxic *E. coli* strain was already observed (Fig. 6A). This effect was significantly less than the one observed at 10 mg ⋅ kg^−1^ DON. We then determined the minimal duration of DON exposure required to exacerbate the genotoxic effect of colibactin. A significant increase in DNA damage in intestinal epithelial cells of rats colonized with *E. coli* WT was observed from 2-week exposure to 10 mg ⋅ kg^−1^ DON, and DNA damage was increased after a 4-week exposure to the food contaminant (Fig. 6B). Taken together, these results indicate that ingestion of a DON-contaminated diet exacerbates the intestinal DNA damage induced by colibactin-producing *E. coli* in a dose- and time-dependent manner.

## DISCUSSION

In the present study, we demonstrated that DON exacerbates the DNA damage induced by *E. coli* producing colibactin both *in vitro* on cultured intestinal epithelial cells and *in vivo* in animals colonized with colibactin-producing *E. coli* and fed DON-contaminated diets.

Several long-term studies showed that DON is not a carcinogenic compound (29, 30); consequently, this mycotoxin has been classified in group 3 (“not classifiable as to its carcinogenicity to humans”) by the World Health Organization (WHO) International Agency for Research on Cancer (IARC). In the present study, DON, used at realistic levels (31, 32), did not induce detectable DNA damage in the intestine. *In vitro*, genotoxicity was observed only upon exposure to very high nonrealistic levels of DON. On the other hand, our *in vitro* and *in vivo* data demonstrated that DON exacerbates the genotoxicity induced by colibactin-producing *E. coli*. This raises questions about the synergism between food contaminants and gut microbiota with regard to intestinal carcinogenesis.

There is overwhelming evidence that DON induces a systemic and intestinal inflammatory response at both the systemic and intestinal levels (33–35). Through this inflammatory effect, DON may predispose the gut epithelium to DNA damage. Indeed, the genotoxic effect of colibactin requires bacterium-host cell contact (9). By decreasing protective mucins and antimicrobial peptide production (36, 37), the inflammation induced by DON could also create an environment in which colibactin-producing *E. coli* bacteria more readily access the epithelium and express their genotoxic potential. This hypothesis has already been proposed to explain the genotoxicity and tumorigenicity of colibactin-producing *E. coli* in azoxymethane-treated IL-10^−/−^ mice (16). Likewise, numerous studies demonstrated that DON induces oxidative stress (38); it stimulates the production of reactive oxygen species (ROS) (39) but has no effect on the production of nitric oxide (40). The rapid generation of ROS was proposed as one of the mechanisms for DNA damage in hepatocytes and lymphocytes exposed to high doses of toxin (41, 42). Secher et al. demonstrated that colibactin-producing *E. coli* strains also trigger the production of intracellular and mitochondrial ROS in infected cells (12), and a recent study shows, in jejunal explants exposed to DON, an increase in the production of COX-2 in the tissue (43). The induction of production of ROS by the mycotoxin and bacteria could explain the exacerbated DNA double-strand breaks in intestinal epithelial cells exposed to both stressors. Finally, DON is also known to activate MAPKs through the phosphorylation of protein kinase R (PKR) (44–46). Recent data suggest that PKR promotes genomic instability by inhibiting DNA damage response signaling and double-strand break repair (47). Increased expression of PKR has also been reported in patients with colon cancer (48). Thus, DON-induced phosphorylation of PKR may exacerbate the genotoxicity induced by colibactin and explain the observed synergism between colibactin and DON. Indeed, in the present study, an increased activation of a MAP kinase was observed in animals colonized by colibactin-producing *E. coli* and exposed to DON.

The prevalence of the specific phylogenetic B2 group, which encompasses *E. coli* strains producing colibactin, is increasing among *E. coli* strains persisting in the microbiota of humans from developed countries (6). DON is the most prevalent fungal toxin present in the food chain in Europe and North America (49, 50). The worldwide incidence of trichothecene contamination and especially of DON has increased in the last years because of climate change, increased use of no-till farming to prevent soil erosion, nonoptimal crop rotations, and inadequate fungicide treatments (51). Since DON is resistant to milling, processing, and heating, this mycotoxin remains present in final food products, such as bread and pasta, obtained from contaminated grain (52). The DON concentrations tested in this study are in accordance with the levels plausibly encountered in the gut after consumption of contaminated food (32). A large percentage of the human population can be exposed to both factors.

In conclusion, our results demonstrate that DON exacerbates the genotoxicity of colibactin. Food contaminants and microbial factors act together to impact host physiology and especially intestinal epithelial cells. This finding raises questions about the interaction between food contaminants and gut microbiota in intestinal carcinogenesis and underlines that the impact of food contaminants, especially mycotoxins, must be evaluated together with the host microbiota.

## MATERIALS AND METHODS

### Bacterial strains and toxins.

*E. coli* bacterial strains (*E. coli* strain M1/5 [14]), bacterial growth conditions, and the use of toxins used in this study are listed in Text S1 and Table S1 in the supplemental material. Purified DON was purchased from Sigma-Aldrich (Saint-Quentin Fallavier, France).

10.1128/mBio.00007-17.1 TEXT S1  Detailed Materials and Methods about bacterial growth conditions, toxins used in immunofluorescence analysis, description of *in vitro* and *in vivo* experimental procedures, analysis of fecal bacterial load, and 16S microbiota analysis in feces from adult animals. Download TEXT S1, DOCX file, 0.02 MB. Copyright © 2017 Payros et al. 2017 Payros et al. This content is distributed under the terms of the Creative Commons Attribution 4.0 International license.

10.1128/mBio.00007-17.5 TABLE S1  Bacterial strains used in this study. Description and genotype of *E. coli* bacterial strains producing colibactin (*E. coli* WT) or not producing colibactin (*E. coli* Δ*clbA* or *E. coli* Δ*clbP*) used in *in vitro* and *in vivo* experiments. Download TABLE S1, PDF file, 0.1 MB. Copyright © 2017 Payros et al. 2017 Payros et al. This content is distributed under the terms of the Creative Commons Attribution 4.0 International license.

10.1128/mBio.00007-17.6 TABLE S2  Composition and mycotoxin contamination of experimental diets. The table shows the composition of the diet and analysis of mycotoxins found in the diet. No mycotoxins were found in the control diet, whereas only DON was found in DON-contaminated diets. Download TABLE S2, PDF file, 0.1 MB. Copyright © 2017 Payros et al. 2017 Payros et al. This content is distributed under the terms of the Creative Commons Attribution 4.0 International license.

### Cell culture.

Nontransformed rat intestinal epithelial IEC-6 cells (ATCC CRL-1592) were cultured as described before (14). Experimental procedures were described in Text S1 and Fig. S1A. At the end of the treatments, in-cell Western (ICW) procedure and immunofluorescence analysis were performed to analyze DNA damage via phosphorylation of the histone H2AX.

The ICW procedure was performed as previously described (53). The cells were fixed, permeabilized, blocked, and then incubated overnight (ON) with rabbit monoclonal anti-γH2AX 1/200 (20E3; Cell Signaling, Saint-Quentin en Yvelines, France). An infrared fluorescent secondary antibody (IRDye 800CW; Rockland) (1/500) was applied simultaneously with RedDot2 (1/500) (Biotium, Interchim, Montluçon, France) for DNA labeling. The DNA and γH2AX were visualized using an Odyssey infrared imaging scanner (LI-COR Science Tec, Les Ulis, France). All experiments were carried out in triplicate.

### Experimental animal model.

Pregnant Wistar female rats (obtained from Janvier Labs, Le Genest Saint-Isle, France) were treated with streptomycin (5 g/liter) and inoculated twice with 10^9^ bacteria by intragastric gavage (14). Animals were given noncontaminated control food or a DON-contaminated diet (10 mg  or 2 mg of DON ⋅ kg of body weight^−1^) for 1 to 4 weeks (Fig. S1B and Text S1).

### Colonic bacterial load and 16S microbiota analysis.

The colonic bacterial load in feces was analyzed before exposure to a DON-contaminated diet at postnatal day 28 (PND 28) and upon completion of the experiment (PND 58). For 16S microbiota analysis, feces were taken at the end of the experiment. Total DNA was isolated from the individual fecal contents using the QIAamp DNA stool minikit (Qiagen, Courtaboeuf, France) (Text S1).

### Histological analysis.

The jejuna fixed in 10% buffered formalin were dehydrated and embedded in paraffin in accordance with standard histological procedures. Sections (5 µm) were stained with hematoxylin-eosin for histopathological evaluation and intestinal morphometry. A lesional score was designed to compare histological changes (44). Images were acquired with a Leica DMRB microscope. Analyses were performed using a MOTIC Image Plus 2.0 image analysis system.

### Immunofluorescence analysis.

Jejunal samples were immediately placed in optimum-cutting-temperature (OCT; Sakura) compound and snap-frozen at −80°C (14). Sections (5 µm) were fixed, permeabilized, blocked with phosphate-buffered saline (PBS) containing 0.1% Tween 20 and 5% normal goat serum (NGS) and stained with rabbit anti-phospho-H2AX antibody (Cell Signaling) followed by Alexa Fluor 546-labeled goat anti-rabbit antibodies (Invitrogen). Slides were mounted in Vectashield containing 4′,6′-diamidino-2-phenylindole (DAPI) (Vector Laboratories). Images were acquired with an Apotome (Zeiss Inc.). γH2AX-positive cells were counted and expressed as a percentage of total epithelial cells. One investigator, blind to the treatment, analyzed all slides.

### Statistical analysis.

All statistical analyses were performed using GraphPad Prism 4.0. The differences between the experimental groups were evaluated using one-way analysis of variance (ANOVA) followed by Bonferroni posttest (which allows for the comparison of all group pairs). All the data were expressed as means ± standard errors of the means (SEM). A *P* value below 0.05 was considered significant.
