# Supplementary figures and images for: The Food Contaminant Deoxynivalenol Exacerbates the Genotoxicity of Gut Microbiota
Source: mBio. 2017 Mar 14;8(2):e00007-17. doi: 10.1128/mBio.00007-17 (PMC5350463; doi:10.1128/mBio.00007-17)

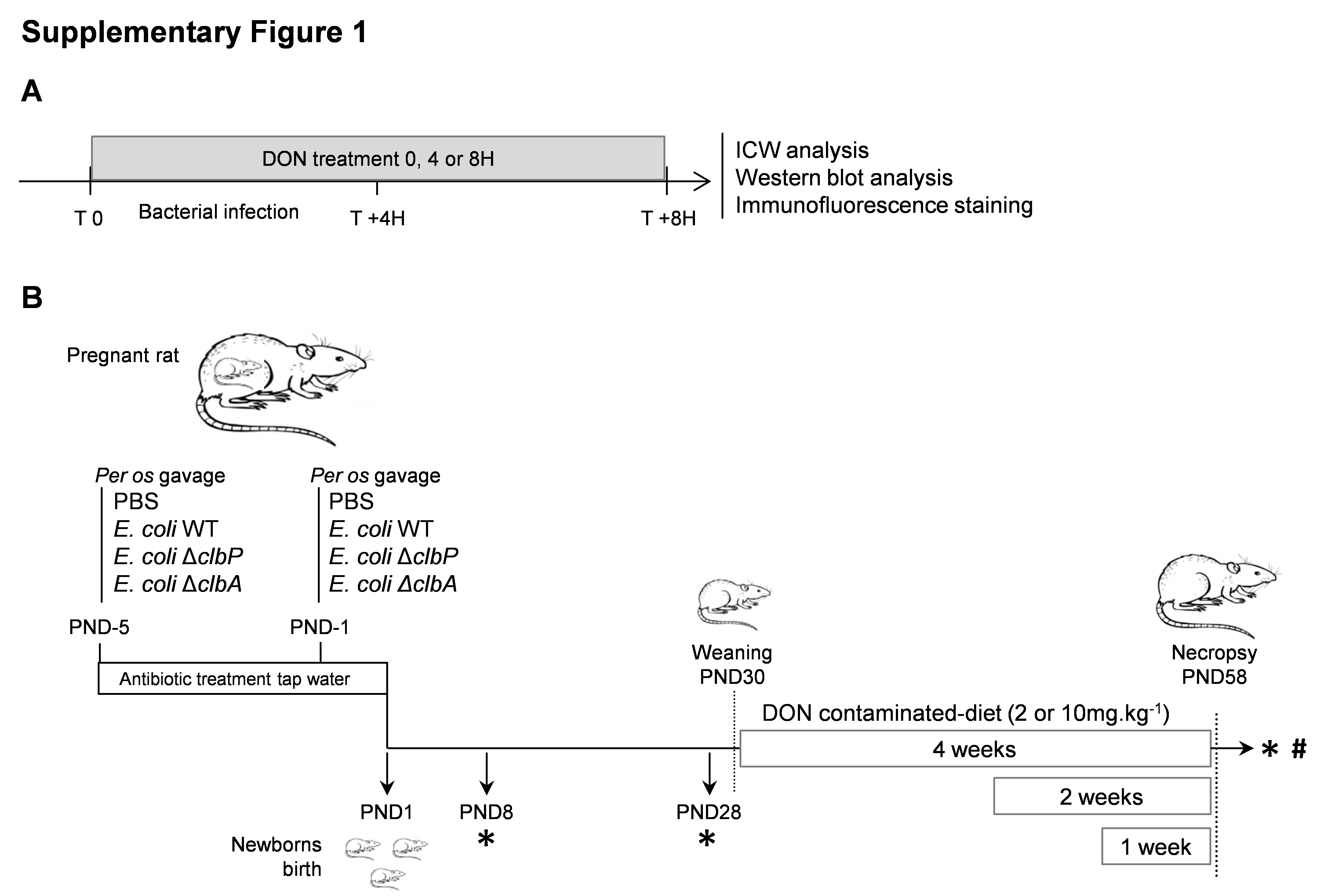

Supplement: FIG S1 [file mbo001173224sf1.tif]
