# Supplementary material for: The Food Contaminant Deoxynivalenol Exacerbates the Genotoxicity of Gut Microbiota
Source: mBio. 2017 Mar 14;8(2):e00007-17. doi: 10.1128/mBio.00007-17 (PMC5350463; doi:10.1128/mBio.00007-17)
Supplement: TEXT S1 [file mbo001173224s1.docx]

**SUPPLEMENTAL INFORMATION**

Food contaminant deoxynivalenol exacerbates the genotoxicity of gut microbiota

**This file includes:**

Supplementary Materials and Methods

Supplementary tables 1 & 2

Supplementary figure 1 to 3 with legend

**Supplementary Materials and Methods**

*Bacterial growth conditions*

For *in vitro* experiments, *E. coli* strains were cultured in Luria-broth (LB) medium overnight (ON) at 37°C with shaking. Specific infection medium (DMEM 25 mM Hepes) was inoculated from ON cultures of *E. coli* in LB medium (1:100 dilution) 2 h at 37°C with shaking (1). Prior to oral administrations, *E. coli* strains were prepared as previously described (1). Bacterial pellets were suspended in sterile PBS to the concentration of 10^9^colony forming units (CFU)/mL.

*Toxins*

Purified DON was purchased from Sigma-Aldrich (Saint-Quentin Fallavier, France). For *in vitro* experiments, DON was dissolved in dimethyl sulfoxide (DMSO). Control samples were treated with DMSO (25µM). For *in vivo* experiments, purified DON was included to animals’diet premix (2). Diet premixes were manufactured at the INRA UPAE (Unité de preparation des aliments, Jouy-en-Josas, France) and formulated according to the requirements established for rats (Table S2).

*In vitro treatments*

IEC-6 cells, used between the 14^th^ and 21^st^ passages, were dispensed in black 96-well cell culture plate (1.5x10^5^ cells/200µL/well). 24 h later, the cells were washed and incubated with increasing doses of DON (0 to 50µM) or/and infected during 4 h with *E. coli* strains (multiplicity of infection (m. o. i.) 0 to 100 bacteria per cell). The cells were then washed and incubated in cell culture medium supplemented with 200µg.ml^-1^ gentamycin. Cells were maintained for a duration of 4 h post infection in presence or not of DON before ICW, western blot or immunofluorescence analysis.

*Immunofluorescence analysis*

Cells were grown and infected on Labtech slides (Falcon). Cells were fixed with PBS 4% formaldéhyde for 20 min, permeabilized in PBS 0.25% Triton X-100 for 15 min and blocked with PBS 0.1% Tween 20 5% normal goat serum for 1 hour. Primary mouse antibody 53BP1 (Interchim, Novus Biological) from rabbit is diluated 1/3000 in blocking solution and incubated ON at 4°C. Alexa fluor 546 from rabbit (Invitrogen) and anti-γH2AX antibody Alexa fluor 488 Conjugate (Millipore) at 1/800 2 hours at RT. Nuclei was stained with DAPI (Molecular Probes) in mounting solution. Confocal laser-scanning microscope (SP8, LEICA) was used to analyzed the slides. The images were analyzed using FIJI software.

*Experimental animal model*

Pregnant rats received streptomycin (5g/L) in tap water and were inoculated twice with 10^9^ CFU of *E. coli* wild-type strain (WT) producing colibactin, *E. coli* ∆*clbA* or *E. coli* ∆*clbP* strains, unable to produce colibactin or treated with PBS (control group) by intragastric gavage before parturition (Post-Natal Day (PND)-5 and PND-1). Animals were sacrificed at PND 8 and PND 58. Animals had free access to water and food. Their body weight was measured on a weekly basis. All animal experiments were performed at the Toxalim animal facility (INRA, UMR 1331, Toulouse), licensed by the French Ministry of Agriculture (agreement n° B31.555.13). All animal experiments complied with the European Union regulation and were reviewed by the Regional Ethics Committee (CNREEA n°1; MP/03/62/11/11).

*Colonic bacterial load*

As previously described (Payros et al. 2014), the colonic bacterial load was analyzed in newborns at post-natal day (PND) 8. As expected, all neonates were equally colonized at PND8 by *E. coli* strains producing or not colibactin. *E. coli* strains levels remained approximately 10^9^ CFU per gram of tissue and represent the species of *Enterobacteriaceae* predominantly found in the newborns’ guts. Before the exposure to DON-contaminated diet at PND28 and upon completion of the experiment (PND58), the colonic bacterial load was analyzed in the feces. Feces homogenates were prepared and 10-fold serial dilution of homogenates was plated on MacCOnkey agar plates supplemented of not with appropriate antibiotics (overnight 37°C). The numbers of CFU were enumerated after 18h. Colonies found growing on agar plates without antibiotic were considered to be *Enterobacteria*-like bacteria belonging to the family *Enterobacteriaceae*.

*16S microbiota analysis*

The V3-4 region of the bacterial 16S rRNA gene was amplified by PCR using the primers recommended by Illumina with overhang adapters and the KAPA HiFi Hot Start Ready Mix (Peqlab, Erlangen, Germany). The PCR products were cleaned using A gencourt AMPure Beads (Beckman Coulter, Krefeld, Germany). A second PCR was performed with the purified DNA to index each of the samples, allowing samples to be pooled for sequencing on one flow cell and subsequently to bed multiplexed for further analysis. The PCR products were a gain cleaned using A gencourt AMPure Beads (Beckman Coulter, Krefeld, Germany). Samples were quality checked and quantified using the SciClone G3 work station (Perkin Elmer) and samples were then pooled in an equimolar fashion. The final 8 pM library containing all pooled samples was run with 5% PhiX samples on the MiSeq sequencer (Illumina, Eindhoven, Netherlands) using a 2 x 300 cycle V3 kit, following standard Illumina sequencing protocols.

*Sequencing Data Analysis*

Sequence reads were processed using mothur version 1.35.0 (Kozich et al. 2013). Chimeric sequences were removed by UCHIME (Edgar et al., 2011) followed by removal of non-bacterial sequences (SILVA119 database, www.arb-silva.de). The remaining sequences were degapped, deuniqued, and split into individual samples. Operational taxonomic unit (OTU) determination was performed by QIIME 1.8.0 (Caporaso et al., 2010) using UCLUST against the Greengenes 13.8 database (DeSantis et al., 2006). An OTU was defined as a group of sequences with a similarity> 97%.
