# Supplementary material for: The Food Contaminant Deoxynivalenol Exacerbates the Genotoxicity of Gut Microbiota
Source: mBio. 2017 Mar 14;8(2):e00007-17. doi: 10.1128/mBio.00007-17 (PMC5350463; doi:10.1128/mBio.00007-17)
Supplement: TABLE S1 [file mbo001173224st1.pdf]

Strains used in the study

E. coli strains	Genotype or phenotype	Source or reference
E. coli WT	M1/5 *E. coli* strain, *rpsL* K42R, streptomycin resistance, carrying *pks* island	Payros et al., 2014
E. coli Δ*clbA*	*rpsL* K42R, streptomycin & kanamycin resistance, *clbA* mutant of strain M1/5	Payros et al., 2014
E. coli Δ*clbP*	*rpsL* K42R, streptomycin & kanamycin resistance, *clbP* mutant of strain M1/5	This study
