# Supplementary material for: The Food Contaminant Deoxynivalenol Exacerbates the Genotoxicity of Gut Microbiota
Source: mBio. 2017 Mar 14;8(2):e00007-17. doi: 10.1128/mBio.00007-17 (PMC5350463; doi:10.1128/mBio.00007-17)
Supplement: TABLE S2 [file mbo001173224st2.pdf]

Supplementary Table S2.

Composition and mycotoxin contamination of experimental diets

	Diet		
	Control	DON 2mg.kg^{-1}	DON 10mg.kg^{-1}
Composition premix			
Cellulose	3.6	3.6	3.6
Caséine chlorhydrique	14.4	14.4	14.4
L Cystine	0.22	0.22	0.22
Mineral compound AIN93G	2.52	2.52	2.52
Vitamin compound AIN93Vx	0.72	0.72	0.72
Bitartrate de choline	0.18	0.18	0.18
Maltodextrine	9.5	9.5	9.5
Maize starch	28.62	28.62	28.62
Sugar	7.2	7.2	7.2
Soya oil	5.04	5.04	5.04
Mycotoxin contamination (mg.kg^{-1})			
Deoxynivalenol	< 0,010	1,75	11,4
3-acetyl DON	n.d.	n.d.	n.d.
15-acetyl DON	n.d.	n.d.	n.d.
Nivalenol	n.d.	n.d.	n.d.
Zearalenone	n.d.	n.d.	n.d.
